# Assessing functional connectivity differences and work-related fatigue in surviving COVID-negative patients

**DOI:** 10.1101/2022.02.01.478677

**Published:** 2022-02-01

**Authors:** Rakibul Hafiz, Tapan Kumar Gandhi, Sapna Mishra, Alok Prasad, Vidur Mahajan, Benjamin H. Natelson, Xin Di, Bharat B. Biswal

**Affiliations:** 1: Department of Biomedical Engineering, New Jersey Institute of Technology (NJIT), 323 Dr Martin Luther King Jr Blvd, Newark, NJ 07102, USA; 2: Department of Electrical Engineering, Indian Institute of Technology (IIT), Block II, IIT Delhi Main Rd, IIT Campus, Hauz Khas, New Delhi, Delhi 110016, India; 3: Internal Medicine, Irene Hospital & Senior Consultant Medicine, Metro Heart and Super-specialty Hospital, New Delhi, India; 4: Centre for Advanced Research in Imaging, Neuroscience & Genomics, Mahajan Imaging, New Delhi, India; 5: Pain and Fatigue Study Center, Department of Neurology, Icahn School of Medicine at Mount Sinai, NY, USA

**Keywords:** COVID, Functional Connectivity, ICA, Fatigue, RS-fMRI

## Abstract

The recent Coronavirus Disease 2019 (COVID-19) has affected all aspects of life around the world. Neuroimaging evidence suggests the novel coronavirus can attack the central nervous system (CNS), causing cerebro-vascular abnormalities in the brain. This can lead to focal changes in cerebral blood flow and metabolic oxygen consumption rate in the brain. However, the extent and spatial locations of brain alterations in COVID-19 survivors are largely unknown. In this study, we have assessed brain functional connectivity (FC) using resting-state functional MRI (RS-fMRI) in 38 (25 males) COVID patients two weeks after hospital discharge, when PCR negative and 31 (24 males) healthy subjects. FC was estimated using independent component analysis (ICA) and dual regression. The COVID group demonstrated significantly enhanced FC in regions from the Occipital and Parietal Lobes, comparing to the HC group. On the other hand, the COVID group exhibited significantly reduced FC in several vermal layers of the cerebellum. More importantly, we noticed negative correlation of FC with self-reported fatigue within regions from the Parietal lobe, which are known to be associated with fatigue.

## Introduction

The novel coronavirus pandemic has taken more than 4.5 million lives across the globe ((WHO), 2020). With efforts of vaccination, mask mandates and social distancing, the spread of this contagious disease has been mitigated significantly, however, advents of new strains such as the delta and omicron variants have been setting back progress and especially affecting densely populated countries (India, being a prime example). While the initial wave demanded most medical attention towards severe damage to the respiratory system, recent evidence suggests the novel coronavirus, Severe Acute Respiratory Syndrome Coronavirus 2 (SARS-CoV-2) can also attack the central nervous system (CNS). Early pandemic MRI reports from acutely ill patients show evidence of a wide range of cerebrovascular abnormalities. A review featuring 22 articles (n = 126 patients) from seven countries (Gulko et al., 2020) showed that patients with SARS-CoV-2 infection showed acute infarcts, posterior reversible encephalopathy syndrome, hyperintensities from fluid-attenuated inversion recovery (FLAIR) images and microhemorrhages. Another multicenter study (n = 64) also reported higher rates of ischemic strokes (27%) and encephalitis (13%). Moreover, inflammatory vascular pathologies (Keller et al., 2020) and other cerebrovascular abnormalities (Nicholson et al., 2020) have also been reported. Abnormal FLAIR uptakes were also reported in the parietal and occipital lobes among others (Kandemirli et al., 2020), as well as in the hypothalamus and the thalamus in individual patients (Paterson et al., 2020).

These single-case reports played a vital role in informing and shaping recent studies with primary focus on group level structural differences in moderate (Duan et al., 2021; Qin et al., 2021) to large sample groups (Douaud et al., 2021). Understandably, the initial target of most neuroimaging studies was brain abnormalities in severe patients. Therefore, a large sample of hospitalized survivors were not investigated, especially those with persistent symptoms. This led to a rise in follow-up studies on a span of 3 to 6 months after initial infection (Lu et al., 2020; Tu et al., 2021) and longitudinal designs (Douaud et al., 2021) where structural abnormalities were investigated before and after the pandemic. We have recently shown gray matter volume differences in survivors after a shorter interval (2 weeks after hospital discharge), as well as, a relation between gray matter volume and self-reported fatigue at work (Hafiz et al., 2021) (in press). It is still unclear, though, if such structural abnormalities are also accompanied by functional brain alterations in COVID-19 survivors.

To address this gap, functional brain imaging can be incorporated through functional magnetic resonance imaging (fMRI). Among the earliest literature, a task-based fMRI study reported loss in task activation in the orbito-frontal cortex (OFC) and strong BOLD activations in the piriform cortex (Ismail & Gad, 2021) from a single female (25 years) COVID patient with persistent olfactory dysfunction. They used a simple smell on/off block design task. A single case resting state fMRI (RS-fMRI) study from an unresponsive patient reported intact functional connectivity (FC) of the default mode network (DMN) (Fischer et al., 2020), which was a good prognosis for ultimate recovery. However, these studies were case reports, which leaves the question of whether there are generalizable group level functional brain alterations in COVID survivors. To that end, a few studies have emerged that report various functional abnormalities among COVID survivors. For example, the initial case report from (Fischer et al., 2020) has now been followed up with a group level report with specific focus on severe patients who were initially unresponsive, but recovered completely and were able to return (Fischer et al., 2021) to pre-COVID level behaviorally. When they compared the functional connectivity of these unresponsive patients with healthy controls, they found significantly reduced default mode network (DMN) connectivity and reduction in inter-network connectivity between DMN and salience (SAL) networks. Currently, a growing concern among survivors is persistence of a sequela of symptoms (Logue et al., 2021; Peluso et al., 2021; Tabacof et al., 2020), now commonly called ‘Long COVID’, which point to brain as the responsible organ. Fatigue, lack of attention, anxiety, memory loss, delayed recovery of smell and/or taste, muscle pain and stress are some of the commonly reported symptoms among many others.

Since several of these symptoms suggest cognitive abnormalities among survivors, most contemporary neuroimaging studies have turned their attention to behavioral correlates of functional brain alterations, primarily, post-traumatic stress syndromes (Benedetti et al., 2021; Fu et al., 2021). On the other hand, several others have attempted to use functional connectivity (FC) as a neurobiological indicator of higher stress levels (Liu et al., 2021; Perica et al., 2021), depression (Zhang et al., 2022) and negative affect (Xiao et al., 2021) among only healthy subjects before and after the pandemic.

Despite fatigue being one of the most frequently reported symptoms, very little is known of functional brain correlates of fatigue within survivors. Prior to the current study, we have shown a positive correlation of gray matter volume within regions from the *ventral basal ganglia (BG)* and *ventromedial prefrontal cortex (vmPFC)* with self-reported fatigue at work in survivors two weeks after hospital discharge (Hafiz et al., 2021) (in press). Here, we continue our investigation to explore the functional correlates of fatigue among the same set of survivors. Moreover, functional changes can occur across different networks among survivors, owing to a range of symptoms experienced during the recovery phase. Therefore, we applied a data driven approach to estimate FC differences between healthy controls (HCs) and surviving, now COVID-negative, patients using RS-fMRI.

The earliest ‘resting state’ study was done by Biswal et al., in 1995 (Biswal et al., 1995), and subsequent studies have shown that spatially distinct regions that are temporally synchronized may share information with each other (Cole et al., 2010; De Luca et al., 2006; Fox et al., 2005; Fox et al., 2006; Greicius et al., 2003; Kalcher et al., 2012; Meier et al., 2012). Independent Component Analysis (ICA), is a data driven technique which groups all voxels in the brain into distinct spatial networks based on the similarity of time courses (McKeown et al., 1998). The large-scale resting-state networks (RSNs) derived from ICA have been shown to have local and higher level associative hierarchy (Yeo et al., 2011) and replicate highly reproducible activation maps across subjects (Smith et al., 2009). FC estimates from group ICA and dual regression (C.F. Beckmann, 2009; Filippini et al., 2009) were used to test our hypothesis that surviving COVID-negative patients would demonstrate altered FC in RSNs comprising of cortical regions where hyperintensities have been reported from single cases and group level differences identified from recent neuroimaging studies. We further hypothesized that FC would demonstrate significant correlation with self-reported fatigue scores in brain regions, known to be associated with fatigue.

## Materials and Methods

### Participants

This is a continuation of our recent publication using the same sample groups where structural brain alterations were reported (Hafiz et al., 2021). 47 COVID patients and 35 healthy controls were recruited from a single site located at Indian Institute of Technology (IIT), Delhi, India. 9 COVID and 4 HC subjects were removed during quality control and motion assessment, leaving with an effective sample of 38 (25 males) COVID and 31 (24 males) HC. The mean age of the COVID group was 34.79 years (SD = ± 10.41 years); and 32.68 years (SD = ± 9.78 years) for the HC group. The COVID subjects were scanned two weeks after they were released from the hospital when confirmed to be COVID-negative upon polymerase chain reaction (PCR) retesting. During scanning, all protocols were strictly followed based on the Institutional Review Board (IRB) guidelines at the Indian Institute of Technology (IIT), Delhi, India.

### Clinical Assessment

The most frequently observed symptoms from the participants during hospitalization were - fever, cough, body ache, chills, difficulty breathing, bowel irritation, nausea, loss of sense of smell and loss of consciousness. From the day of discharge till the day of scan, we further asked if the participants were experiencing any persistent or new symptoms. Work-related fatigue (65.6%), muscle pain (50%), lack of sleep (50%), lack of attention (43.8%), headache (40.6%), joint pain (40.6%), memory loss (28.1%), delayed recovery of sense of smell (39%) and/or taste (31%), bowel irritation (33%) and interestingly, hair loss (66%) were commonly reported. Please note, most survivors experienced multiple symptoms simultaneously, hence the ‘%’ represents symptoms that overlap within participants. For example, 43.8% of 27 post-COVID participants reporting with lack of attention also reported a work-related fatigue score > 2 on a scale of 0 to 5, with 0 representing no fatigue and 5 representing the highest fatigue possible. The average fatigue score in this sub-set of COVID participants was 2.93/5 ± 1.21 [SD].

### Brain Imaging

#### Anatomical MRI –

High-resolution T1-weighted images were acquired on a 3T GE scanner with a 32-channel head coil in 3D imaging mode with a fast BRAVO sequence. The imaging parameters were TI = 450 ms; 244 × 200 matrix; Flip angle = 12 and FOV = 256 mm. The subject was placed in a supine position and the whole brain was scanned in the sagittal configuration where 152 slices were collected, and each slice was 1.00 mm thick. The spatial resolution of all the anatomical scans was 1.0 mm × 1.0 mm × 1.0 mm.

#### Resting-state fMRI –

A gradient echo planar imaging (EPI) was used to obtain 200 whole-brain functional volumes. The parameters were: TR = 2000 ms; TE = 30 ms; Flip angle = 90, 38 slices, matrix = 64×64; FOV = 240 × 240 mm^2^; acquisition voxel size = 3.75 × 3.75 × 3 mm^3^. The participant was requested to stay as still and motionless as possible with eyes fixed to a cross on an overhead screen.

### Data Pre-Processing

The data preprocessing was performed primarily using Statistical Parametric Mapping 12 (SPM12) toolbox (http://www.fil.ion.ucl.ac.uk/spm/) within a MATLAB environment (The MathWorks, Inc., Natick, MA, USA). However, some steps utilized useful tools from FSL (FMRIB Analysis Group, Oxford, UK) and AFNI (http://afni.nimh.nih.gov/afni) (Cox, 1996) for housekeeping, visual inspection and quality control purposes. At the beginning, first five time points were excluded from each subject to account for magnetic stabilization. The functional images were motion corrected for head movement using a least squared approach and 6 parameters (rigid body) spatial transformation with respect to the mean image of the scan. The subjects with excessive head motion were identified using framewise displacement (FWD) (Power et al., 2012). Additionally, time frames with high FWD crossing a threshold of 0.5 mm (Power et al., 2012) were identified along with the previous and the next two frames and added as regressors (Yan et al., 2016) during temporal regression of nuisance signals. If more than 50% of the time series data were affected due to regression of high motion frames the participant was removed from the analysis. Moreover, any participant with the maximum framewise translation or rotation exceeding 2 mm was removed from further analysis. Anatomical image from each subject was coregistered to the mean functional image obtained from the motion correction step.

T1-weighted image from each subject was segmented into gray matter (GM), white matter (WM), and cerebrospinal fluid (CSF) tissue probability maps and an average template including all participants was generated using DARTEL (Ashburner, 2007). This template was used to spatially normalize all functional images to the MNI space and resampled to isotropic voxel size of 3 mm × 3 mm × 3 mm. Time series, from brain compartments with high physiological noise signals such as, CSF and WM was extracted by thresholding the probability maps from the segmentation stage above the 99^th^ percentile, and first 5 principial components were obtained using a COMPCOR based (Behzadi et al., 2007) principal component analysis (PCA) from both tissues. These 10 components along with Friston’s 24- parameter model (6 head motion parameters + 6 previous time point motion parameters + 12 corresponding quadratic parameters) (Friston et al., 1996) and time frames with high FWD (> 0.5 mm) were added as regressors in a multiple linear regression model to remove unwanted signals voxel-wise. The residuals from the regression step were then bandpass filtered between 0.01 to 0.1 Hz and finally, spatial smoothing was performed using a Gaussian kernel of 6 mm full width at half maximum (FWHM).

### Head Motion Assessment

We performed in-scanner head movement assessment using mean Framewise Displacement (FWD) based on the methods depicted in (Power et al., 2012). A two-tailed two-sample student’s t-test revealed no significant differences in mean FWD between the two groups (*t = −1.57, p = 0.12, α = 0.05*).

### ICA and Dual Regression

Group level resting state networks were obtained by applying the ‘gica’ option of the ‘melodic’ module from FSL toolbox (FMRIB Analysis Group, Oxford, UK). All subjects’ 4D functional images after pre-processing were temporally concatenated into a 2D matrix of ‘space’ × ‘time’ as delineated in (C.F. Beckmann, 2009) and 25 spatial maps were obtained. Resting State Networks (RSNs) were identified by matching ICs with the 1000 functional connectome project maps (Biswal et al., 2010) using Dice’s coefficient and spatial correlations obtained from AFNI’s ‘3dMatch’ program (Taylor & Saad, 2013). Further visual inspection was performed to make sure all network regions aligned with the functional network and ROIs depicted in (Altmann et al., 2015; Shirer et al., 2012). Dual regression (C.F. Beckmann, 2009; Filippini et al., 2009) was performed leveraging the standardized group ICA output from the ‘melodic’ step and applying it directly to the ‘fsl-glm’ module in FSL to obtain subject specific RSN maps. The subject specific network maps were standardized to Z-scores before consequently applying them in statistical analysis to infer group level estimates.

### Statistical Analysis

To investigate FC differences between COVID and HC groups, we performed an unpaired two sample t-test between standardized subject-specific RSN maps from the two groups. Significant clusters were identified and main effect of interest from the corresponding contrast maps representing the difference in mean beta scores from two groups were obtained by thresholding the t-score map values that survived the corrected threshold. To account for confounding effects that may explain some of the variance in the data, age and sex were also added as covariates of no interest. Cluster-based thresholding was applied at height threshold of *p*_*unc*_ < *0.01*, with *family wise error* (*FWE)* correction at *p*_*FWE*_ < *0.05* for multiple comparisons. The cluster extent threshold (*k*_*E*_) obtained from this step was used to generate corrected statistical maps for the contrasts with significant effects.

We further wanted to evaluate which of the large scale RSNs demonstrates linear relationship or correlation with self-reported fatigue among the COVID individuals. We incorporated a multiple linear regression approach where the FC at each voxel was the response variable (Y), and the self-reported fatigue score was the explanatory variable (X). We also added age and sex as covariates of no interest. Significant clusters were obtained in the same manner as described earlier in this section for group level differences in FC. For visual representation of the significant linear relationship between the two variables, the average FC within the significant cluster was obtained from each subject. These average FC values were then linearly regressed against the fatigue scores and visualized within a scatter plot and a line of best fit with 95% confidence interval. Age and sex were regressed out during the linear regression step. The correlation analysis and the graphical plotting was done using ‘inhouse’ scripts prepared in RStudio (RStudio, 2021).

## Results

**Figure 1.**
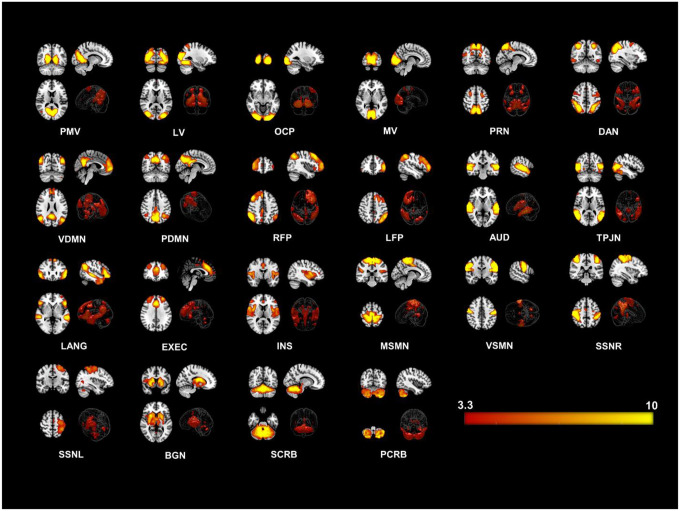
Twenty-two Resting State Networks (RSNs) identified from group ICA using ‘melodic’. Abbreviated names of each network are shown at the bottom of each image. Three orthogonal slices are shown for each network along with a volume rendered image to show depth and three-dimensional view of the RSNs. Statistical estimates (Z-scores) are embedded into a colorbar at the bottom-right. PMV = Primary Visual Network, LV = Lateral Visual, OCP = Occipital Pole, MV = Medial Visual, PRN = Precuneus Network, DAN = Dorsal Attention, VDMN = Ventral Default Mode Network (DMN), PDMN = Posterior DMN, RFP = Right Fronto Parietal, LFP = Left Fronto Parietal, AUD = Auditory, TPJN = Temporo-Parietal Junction Network, LANG = Language Network, EXEC = Executive Control Network, INS = Insular Network, MSMN = Medial Sensory-Motor Network (SMN), VSMN = Ventral SMN, SSNR = Somatosensory Network - Right, SMNL = Somatosensory Network - Left, , BGN = Basal Ganglia Network, SCRB = Superior Cerebellar Network, PCRB = Posterior Cerebellar Network.

**Figure 2.**
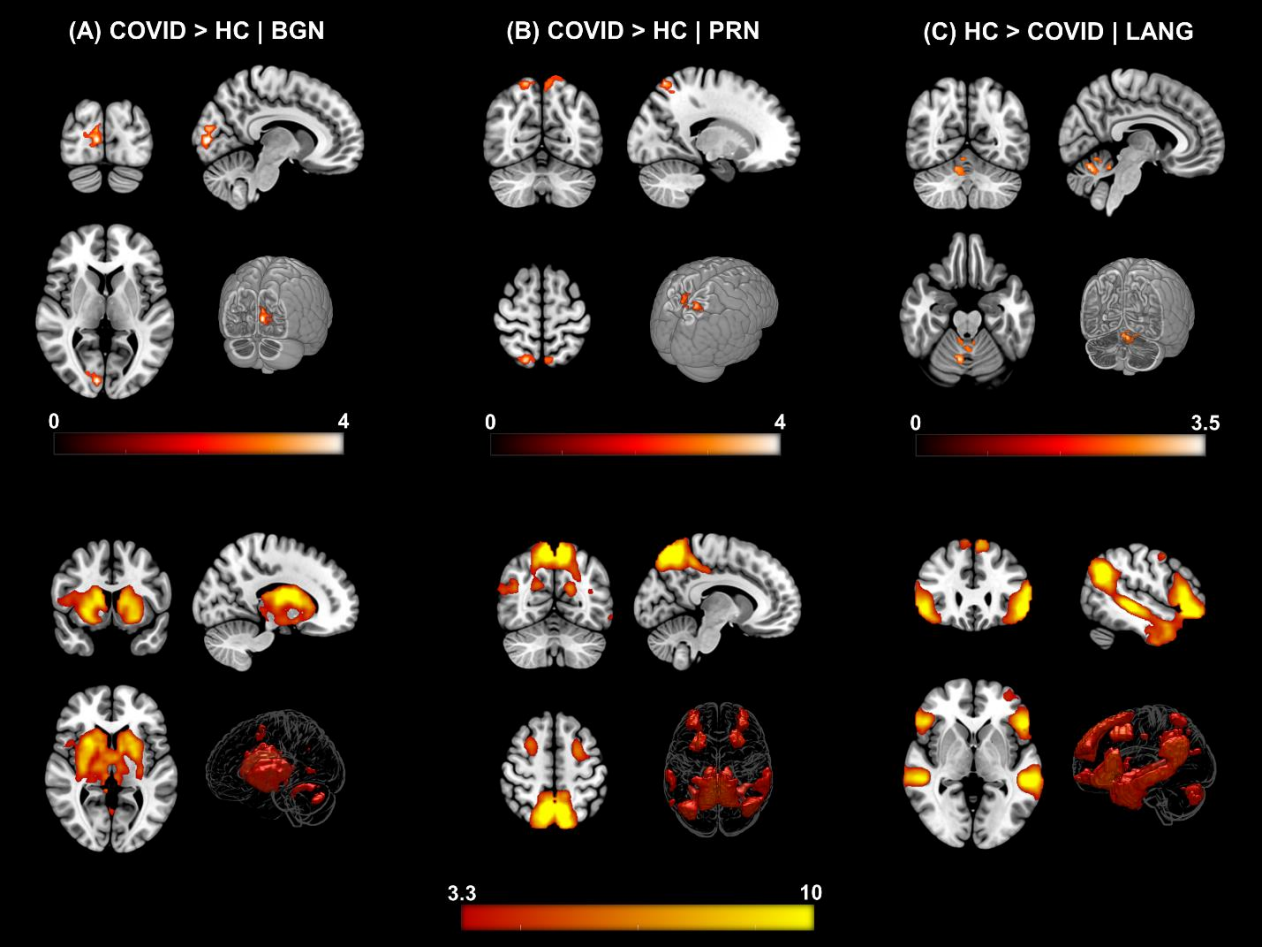
ΔFC | Functional Connectivity differences between COVID survivors and healthy controls. **[top row] (A) COVID > HC:** Enhanced FC in COVID compared to HCs observed in the *Basal Ganglia Network (BGN)* network. Three orthogonal slices (left) along with a cut-to-depth volume rendered image to show the effects in the *right Calc, Cu* and *LiG*. The colorbar represents *t* – score values. Cluster information include - cluster peak: *[9 −84 6]*, | cluster extent threshold, *k*_*E*_ = *69* | cluster size = *69 voxels*. **(B) COVID > HC:** Enhanced FC in COVID compared to HCs observed in the *Precuneus (PRC)* network, demonstrating a significant difference in FC in the *bilateral SPL and PCu* regions. Cluster information include - cluster peak: *[21 −57 54]*, | cluster extent threshold, *k*_*E*_ = *90* | cluster size = *90 voxels***. (C) HC > COVID:** Enhanced FC in HCs compared to COVID observed in the *Language (LANG)* network demonstrating significant difference in several vermal layers of the *Cerebellum*. Cluster information include - cluster peak: *[9 −63 −24]*, | cluster extent threshold, *k*_*E*_ = *57* | cluster size = *57 voxels*. [**bottom row]** Corresponding group level ICA networks from which FC differences are shown on the top row. The colorbar represents z-scores.

**Figure 3.**
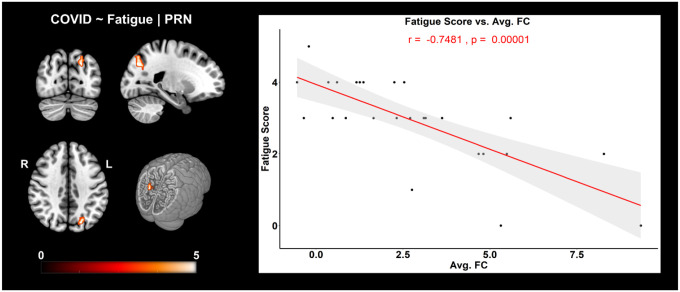
FC corr. Fatigue | COVID: Negative correlation of FC with self-reported fatigue scores in COVID individuals. **(left)** For the *PRN* network, three orthogonal slices (left) along with a cut-to-depth volume rendered image showing regions from the *Superior Parietal* and *Occipital* Gyri that demonstrated significantly negative correlation with fatigue. The colorbar represents t-score values. **(right)** The graph shows the linear relationship of the average FC within the significant cluster and self-reported fatigue scores from COVID individuals. The x-axis represents the average FC (z-scores) from the cluster and the y-axis represents the fatigue scores. The shaded gray area represents the 95% confidence interval. The red line represents the least squares regression line of best fit. Cluster information include - cluster peak: *[−21 −75 36]*, | cluster extent threshold, *k*_*E*_ = *58* and cluster size = *58 voxels*.

We identified twenty-two large-scale resting state networks (RSNs) (see Figure1) from the group ICA analysis. Group level statistical analysis was run for each network using standardized subject specific RSN maps obtained from the dual regression step. Significantly enhanced FC was observed in the COVID group compared to the HC group in particularly regions from the *occipital* and *parietal* lobes. Figures2 and 3 show all significant clusters from the FC and linear regression analysis, respectively.

Figure2 shows the results from the group level analysis from three RSNs. Figure2 A (top row) demonstrates regions with significantly enhanced FC in the COVID-19 group compared to the HC group for the *BGN* network. The FC difference was observed in the *Right – Calcarine Cortex (Calc), Cuneus (Cu)* and *Lingual Gyrus (LiG)* regions of the *occipital* lobe. Similarly, the COVID survivors also demonstrated enhanced FC of the *PRN* network (Figure2 B) with regions from the *Parietal Lobe*: *Bilateral – Superior Parietal Lobule (SPL) and Precuneus (PCu)* regions. On the other hand, Figure2 C shows reduced functional connectivity among COVID participants compared to HCs in several layers of the *cerebellar vermal lobules (CVL) (I-V, VI-VII)* for the *LANG* network. The cluster peak information including peak T-scores and *FWE* corrected exact p-values with relevant spatial regions from each network showing significant differences have been tabulated for an easy reference in Table1.

Figure3 shows brain regions where a significant negative correlation was observed between FC and self-reported fatigue, from the *PRN* network. Figure3 (A) (left) shows the cluster where a negative correlation between FC and fatigue scores was observed in the *Left – Superior Parietal Lobule (SPL), Superior Occipital Gyrus (SOG), Angular Gyrus (AnG)* and *Precuneus (PCu)*. The graph on the right visually presents this negative relationship (*r = −0.75, p = 0.00001, r*^*2*^ = *0.56*) between the average FC of this cluster and fatigue scores.

## Discussion

The results from this study support our hypothesis that COVID survivors would demonstrate altered FC when compared to HCs, even two weeks after discharge from the hospital and demonstrate significant linear relationship with work-related fatigue. Our hypothesis was based on both early case-reports and more recent group level neuroimaging reports of structural and functional brain alterations. Individual case reports were primarily from acutely ill patients using FLAIR (Kandemirli et al., 2020; Kremer et al., 2020; Paterson et al., 2020) and Susceptibility Weighted Imaging (SWI) (Conklin et al., 2021), whereas, group level reports, such as those derived from fMRI, include, reduced *default mode* and *salience* connectivity (Fischer et al., 2021) and high prevalence of abnormal time varying and topological organizations between *sensorimotor* and *visual* networks (Fu et al., 2021). In the current context, we report between group FC alterations of three large scale RSNs – *BGN*, *PRN* and *LANG* networks and further show negative correlation of FC from the *PRN* network with self-reported fatigue at work among COVID survivors.

While initially a single patient showed no differences in FC of *DMN* when compared to five healthy controls (Fischer et al., 2020), Fischer and colleagues recently reported reduced FC within *DMN* and between *DMN* and *SAL* networks after group level assessment (Fischer et al., 2021). In the current study, we did not observe any significant alterations in posterior or ventral DMN (PDMN and VDMN) networks, but our patient group was not unresponsive and as acutely ill as the patients reported in (Fischer et al., 2021). However, we did observe differences in FC for the PRN network which consists of *Precuneus (PCu), Frontal Eye Fields (FEF)* and parts of the *Superior Parietal Lobule (SPL)*. Enhanced FC in this network was observed in the *Bilateral SPL* and *PCu* regions*. PCu* is a constituent of DMN, and higher functional connectivity with this region may indicate some compensatory mechanism due to loss in connections in other pathways. Furthermore, *SPL* is a constituent of the *posterior parietal cortex (PPC)* which has been shown to have functional association with altered *anterior insula* connectivity in chronic fatigue syndrome (CFS) (Wortinger et al., 2017). Moreover, these brain regions are also known to be involved in attention processing, therefore, enhanced FC in these regions may indicate possible compensatory mechanisms of attention related symptoms that recovering patients may experience. Therefore, further investigations are necessary to understand these processes better, especially, from a clinical perspective.

Enhanced FC in the COVID group was also observed for the *BGN* network within the occipital lobe (*Calc, Cu* and *LiG*). *Calc* and *Cu* are primarily involved in visual processing. Fu and colleagues reported that COVID survivors had higher connectivity between *Cerebellum*, *Sensorimotor* and *Visual* networks, indicating they spent abnormally higher time in a specific brain state compared to healthy controls (Fu et al., 2021). A recent study has also suggested *Cu* to be a major hub for mild cognitive impairment in idiopathic REM sleep behavior disorder (iRBD) (Mattioli et al., 2021). *LiG* and weak *insular* coactivation with the *occipital* cortex have been shown to be associated with disrupted salience processing that can lead to loss in motivation in day-to-day tasks (Kim et al., 2018).

Moreover, the *basal ganglia* are known to be associated with fatigue (Miller et al., 2014), cognitive, emotional and attention processing (Di Martino et al., 2008; van Schouwenburg et al., 2015). We also observed reduced FC within several layers of the *Cerebellar Vermal Lobules* among COVID participants when compared to HCs. These lobules have been suggested to be involved in cognition and emotion processing (Park et al., 2018). The synergy of these studies to our findings indicates possible functional brain associations of commonly observed symptoms in survivors with post-acute sequelae SARS-CoV-2 infection (PASC or Long COVID) lasting many months (Carfì et al., 2020; Garrigues et al., 2020; Logue et al., 2021; Peluso et al., 2021). FC alterations in multiple networks also suggest that RS-fMRI can be quite useful to investigate multiple brain networks across the whole brain (Damoiseaux et al., 2006; Raichle & Mintun, 2006; Shulman et al., 2004) in COVID-19 patients. The results also suggest a possible link between structural and functional abnormalities in COVID patients since the FC alterations were observed in regions that align with anatomical regions exhibiting hyperintensities from FLAIR and SWI studies.

We further evaluated linear relationship between FC of RSNs and self-reported fatigue at work among COVID participants. We observed a significant negative correlation of FC with fatigue within the *Left SPL, SOG, AnG* and *PCu*, i.e., brain regions primarily belonging to the *parietal* lobe (see Table2 for cluster information). Structural atrophy in the *parietal* lobe has been shown to be associated with fatigue among multiple sclerosis (MS) patients (Calabrese et al., 2010; Pellicano et al., 2010). An RS-fMRI study of patients with chronic fatigue syndrome (CFS) used ICA to reveal loss of intrinsic connectivity in the parietal lobe (Gay et al., 2016). It is interesting that lower FC in the parietal lobe correlates negatively to higher fatigue scores among COVID survivors. To the best of our knowledge, this is the first study to show work-related fatigue correlates of FC among recovering patients 2 weeks after hospital discharge. Therefore, future studies are necessary to evaluate this avenue further in the surviving cohorts.

## Limitations

Despite our efforts to show group level effects that reflect individual and group level reports in the recent literature, our study still maintains a cross-sectional design. In cases like this, a better approach for the future would be to use follow up designs (Fu et al., 2021; Lu et al., 2020; Tu et al., 2021) or possibly a longitudinal design where patients could be observed both before and after the pandemic like the one using the UK-biobank (Douaud et al., 2021). Our effort here, was to show group level effects at an early stage of recovery (2 weeks after hospital discharge) and determine the relation between work-related fatigue and FC of RSNs. We believe the results from this study will help understanding the recovery stage brain alterations and how they might drive fatigue-related symptoms among COVID survivors.

## Figures and Tables

**Table 1.** List of spatial regions from significant clusters obtained from the contrast – COVID > HC.

ΔFC	RSN Name	Cl. No.	Anatomical Locations	Cl. Ext.	Cl. Size	Peak MNI Coordinates			Peak T, p_FWE_ Values
						X	Y	Z	
COVID > HC	*BGN*	1	*Right – Calcarine Cortex (Calc)*	69	69	9	−84	6	4.46, 0.004
			*Right – Cuneus (Cun)*						
			*Right – Lingual Gyrus (LiG)*						
	*PRN*	1	*Right – Superior Parietal Lobule (SPL)*	90	90	21	−57	54	4.22, 0.001
			*Right – Precuneus (PCu)*						
			*Left – Superior Parietal Lobule (SPL)*						
			*Left – Precuneus (PCu)*						

HC > COVID	*LANG*	1	*Right – Cerebellar Exterior (CExt.)*	57	57	9	−63	−24	3.56, 0.019
			*Right – Cerebellar Vermal Lobules I–V*						
			*Right – Cerebellar Vermal Lobules VI–VII*						

The regions from three RSNs – *BGN*, *PRN* and *LANG* which demonstrated significant differences are presented with peak MNI coordinates (X Y Z) and corresponding peak t-score values for each cluster. Keys – ΔFC = Direction of change in Functional Connectivity; Cl. = Cluster; Cl. No. = Cluster Number; Cl. Ext. = Cluster Extent Threshold; Cl. Size = Cluster Size; T = peak t-score; p_FWE_ = corrected p-value.

**Table 2.** List of spatial regions from clusters showing significant correlation with self-reported fatigue among COVID individuals.

Corr (FC, ftg.)	RSN Name	Cl. No.	Anatomical Locations	Cl. Ext.	Cl. Size	Peak MNI Coordinates			Peak T, p_FWE_ Values
						X	Y	Z	
COVID	*PRN*	1	*Left – Superior Parietal Lobule (SPL)*	58	58	−21	−75	36	5.31, 0.01
			*Left – Superior Occipital Gyrus (SOG)*						
			*Left – Angular Gyrus (AnG)*						
			*Left – Precuneus (PCu)*						

The regions from *PRN* which demonstrated significant correlation are presented with peak MNI coordinates (X Y Z) and corresponding peak t-score values for each cluster. Keys – FC = Functional Connectivity; ftg. = Fatigue Scores, Cl. = Cluster; Cl. No. = Cluster Number; Cl. Ext. = Cluster Extent Threshold; Cl. Size = Cluster Size; T = peak t-score; p_FWE_ = corrected p-value.

## Significance Statement

Early neuroimaging studies have mostly focused on structural MRI imaging to report brain abnormalities in acutely ill COVID-19 patients. It is not clear whether functional abnormalities co-exist with structural alterations in patients who have survived the infection and have been discharged from the hospital. A few recent studies have emerged which attempted to address the structural/functional alterations. However, further investigations across different sites are necessary for more conclusive inference. More importantly, fatigue is a highly prevalent symptom among COVID survivors, therefore, the relations of brain imaging abnormalities to fatigue should be investigated. In this study, we try to address these gaps, by collecting imaging data from COVID survivors, now PCR negative, and healthy subjects from a single site – the Indian Institute of Technology (IIT), Delhi, India. Furthermore, this is a continuation of an ongoing study. We have already submitted a manuscript showing structural abnormalities and gray matter volume correlates of self-reported fatigue among this group of COVID survivors.
